# Spin‐Polarized Interfaces Redirect CO_2_ Reduction From CO to Formate

**DOI:** 10.1002/anie.1729522

**Published:** 2026-08-20

**Authors:** Fan He, Xingxu Yan, Xiaoqing Pan, Yong Yan, Jing Gu

**Affiliations:** ^1^ Department of Chemistry and Biochemistry San Diego State University San Diego USA; ^2^ Department of Materials Science and Engineering University of California Irvine USA; ^3^ Department of Physics and Astronomy University of California, Irvine Irvine USA

**Keywords:** carbon dioxide reduction, chiral‐induced spin selectivity, formate selectivity, intermediate formation

## Abstract

Achieving high product selectivity in electrocatalytic carbon dioxide reduction (CO_2_RR) remains a critical challenge due to competition between multiple proton‐coupled electron‐transfer pathways on catalyst surfaces. Meanwhile, chirality‐induced spin selectivity (CISS), which enables spin‐polarized electron transport through chiral interfaces, has recently emerged as a promising strategy to modulate interfacial electrochemical reactions. Although the CISS effect has been shown to enhance selectivity and efficiency in the spin‐sensitive oxygen evolution reaction (OER), its role in regulating CO_2_RR pathways and in stabilizing intermediates remains largely unexplored. Here, chiral molecules (R‐ and S‐1,1′‐bi‐2‐naphthyl‐2,2′‐diyl hydrogen phosphate, BNP) were integrated with SnO_2_ to construct chiral‐modified catalysts (R‐BNP/SnO_2_ and S‐BNP/SnO_2_). Compared with bare SnO_2_ and racemic BNP‐modified SnO_2_ (Rac‐BNP/SnO_2_), the chiral catalysts exhibited a pronounced shift in product selectivity from CO toward formate production. Importantly, in‐situ attenuated total reflectance surface‐enhanced infrared absorption spectroscopy (ATR‐SEIRAS) reveals that the chiral interface selectively stabilizes the O‐bound *OCHO intermediate associated with the formate pathway and modulates interfacial water structure and hydrogen‐bonding dynamics. These findings demonstrate that spin‐polarized interfacial electron transfer can regulate CO_2_RR pathway selectivity by modulating the stabilization of key intermediates. More broadly, this work establishes chiral spin‐selective interfaces as a new strategy for regulating competitive electrocatalytic reaction pathways.

## Graphical abstract

The chiral electrocatalyst exploits the CISS effect to establish a spin‐polarized interface that selectively promotes formate production during CO_2_ reduction while effectively suppressing the competing hydrogen evolution.

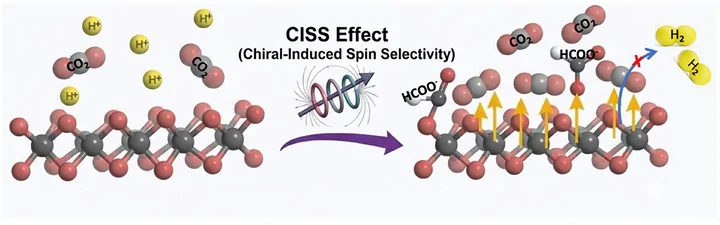

## 1 Introduction

Electrochemical carbon dioxide reduction (CO_2_RR) offers a promising route to convert greenhouse gases into valuable fuels and chemicals using renewable electricity. Despite significant progress in catalyst design, the selective conversion of CO_2_ into value‐added chemicals remains a grand challenge, especially for reaction pathways with nearly identical thermodynamics [1, 2, 3]. CO_2_RR exemplifies a prototypical selectivity problem, in which multiple proton‐coupled electron transfer (PCET) steps branch into a diverse range of C_1_ and C_2_ products [4, 5, 6, 7, 8]. Conventional approaches, such as tuning surface composition, crystal facet, or local reaction environments, have led to incremental improvements but are fundamentally constrained by the scaling relationships inherent to classical thermochemistry [9, 10, 11, 12]. Consequently, a conceptual leap is needed: beyond optimizing adsorption energies alone, precise control over selectivity requires altering the electron‐spin character of interfacial electron‐transfer steps without a proportional change in adsorption strength [13].

In heterogeneous catalysis, product distributions are traditionally interpreted through the Sabatier principle [14, 15], which states that optimal catalytic performance is achieved when reaction intermediates bind to the catalyst surface with neither excessively weak nor overly strong interactions. In spin‐sensitive catalytic systems, this adsorption energy landscape can be further modulated by the electron‐spin configuration of the catalyst and reactants, providing an additional handle on selectivity. However, deliberate spin control as a strategy to overcome scaling‐relation constraints remains underexplored. Contemporary approaches are still predominantly non‐spin‐polarized, even though intermediate branching, such as the competition between *COOH (carboxyl‐type) and *OCHO (formate‐type) pathways, leading to CO (E^o ^= −0.52 V) and HCOOH (E^o ^= −0.61 V), respectively, is fundamentally governed by subtle differences in intermediate stabilization and reaction kinetics [16, 17]. Such open‐shell intermediates provide plausible pathways through which spin‐polarized electrons may influence reaction kinetics and intermediate stabilization, consistent with previous studies of spin‐dependent electron transfer and catalytic processes [18, 19]. In this regard, spin polarization offers a fundamentally underexplored degree of freedom for modulating intermediate energetics and steering catalytic reaction pathways.

Among the few routes available to impose spin polarization at catalytic interfaces, the Chirality‐Induced Spin Selectivity (CISS) effect is especially attractive because it converts molecular chirality into spin‐filtered electron transport [20, 21, 22, 23]. In chiral or helical potentials with strong spin–orbit coupling, electron transport becomes spin‐selective via spin–momentum locking, generating a spin‐polarized electron flux at the catalyst–electrolyte interface [24, 25, 26]. This introduces a kinetic bias toward reaction pathways that are more compatible with the spin state of the transient intermediates. While CISS has been leveraged in photocatalysis and oxygen evolution to modulate singlet–triplet product distributions [27, 28, 29, 30], its application to CO_2_RR presents greater mechanistic complexity and opportunity.

By grafting chiral 1,1′‐bi‐2‐naphthyl‐2,2′‐diyl hydrogen phosphate (BNP) molecules onto SnO_2_, we show that chirality serves not only as a structural modifier but also as an interfacial regulator, redirecting CO_2_RR pathways. The chiral interface promotes formate selectivity by stabilizing the O‐bound *OCHO intermediate while suppressing the competitive HER. In‐situ attenuated total reflectance surface‐enhanced infrared absorption spectroscopy (ATR–SEIRAS) provides direct evidence that a chiral modification shifts the dominant reaction pathway from *COOH‐mediated CO formation to *OCHO‐mediated formate production. In addition, ATR–SEIRAS shows that chiral interfaces disrupt the hydrogen‐bond network and constrain water reorientation, providing a molecular‐level understanding of how CISS effects suppress HER kinetics. By coupling chiral molecular engineering with electrocatalysis, this strategy introduces spin‐polarized interfacial electron transfer as a new dimension for tuning catalytic selectivity and steering competitive multi‐electron reaction pathways.

## 2 Results and Discussion

### 2.1 Chiral Electrocatalyst Fabrication and Characterization

We fabricated SnO_2_ by using Sn‐terephthalic acid metal‐organic frameworks (SnBDC) as a precursor, followed by annealing in air. Afterward, R‐, S‐, and Rac‐BNP were mixed with as‐prepared SnO_2_ to form a chiral or racemic catalyst, denoted as R‐BNP/SnO_2_, S‐BNP/SnO_2_, and Rac‐BNP/SnO_2_, respectively (Figure 1a). BNP is an atropisomeric binaphthyl‐derived Brønsted acid with broad applications in asymmetric catalysis, chiral recognition and separation, and supramolecular and interfacial chemistry [31, 32, 33]. In this study, BNP was selected for several reasons. First, its phosphate functionality enables strong coordination with Sn sites on SnO_2_, forming stable Sn─O─P bonds [34, 35] (Figure S1). Second, the conjugate binaphthyl backbone facilitates electronic coupling across the inorganic‐organic interface without significantly impeding conductivity [36]. Third, the electron‐deficient nature of aromatic rings increases their redox stability [37]. In addition, it is worth mentioning that since BNP's chirality comes from the axial chirality of its binaphthyl backbone, the closest achiral control is the racemic mixture of BNP [38]. Figure 1b presents a high‐resolution scanning transmission electron microscopy (STEM) image of R‐BNP/SnO_2_. In contrast to bare SnO_2_ (Figures S2 and S3), a discontinuous layer of BNP molecules is observed on the SnO_2_ surface, while the crystalline lattice of SnO_2_ remains clearly visible. The lattice fringe with a spacing of 0.34 nm [39], corresponding to the (110) plane of SnO_2_, is well preserved, indicating that BNP modification does not alter the crystal structure of SnO_2_. Importantly, the discontinuous molecular layer leaves some regions of the SnO_2_ surface exposed, providing direct access of CO_2_ molecules and electrolyte species to the catalytically active Sn sites. The discontinuous layer of BNP indicates that BNP does not serve as an electron‐transport medium; rather, it functions as an interfacial chiral modifier that regulates the local electronic environment and spin‐dependent electron transfer at the catalyst/electrolyte interface. Moreover, the energy‐dispersive x‐ray spectroscopy (EDS) images showed a homogeneous distribution of Sn, O, C, and P (Figures 1c and S3). The successful synthesis of SnO_2_ is further confirmed by Raman spectroscopy and Xx‐ray diffraction (XRD) pattern. The Raman spectra of pristine and chiral, racemic‐molecule‐decorated SnO_2_ exhibit characteristic bands at 474, 632, and 775 cm^−1^, corresponding to the E_g_, A_1 g_, and B_2 g_ vibrational modes (Figure 1d), respectively, which arise from symmetric and asymmetric stretching of Sn─O [40]. The diffraction peaks located at 26.7°(110), 33.9°(101), 38.0°(200), 51.8°(220), 54.8°(301), 66.1°(301) for all SnO_2_ samples are in a good agreement with tetragonal SnO_2_ phase (PDF#41‐1445) [41] (Figure 1e). Following deposition of chiral and racemic molecular layers, Raman spectra and XRD patterns of R‐BNP/SnO_2_, S‐BNP/SnO_2_, and Rac‐BNP/SnO_2_ remain essentially unchanged, indicating that BNP incorporation does not alter the crystal structure of pristine SnO_2_. To verify the existence of the chiral molecule and study the interaction between BNP and SnO_2_, x‐ray photoelectron spectroscopy (XPS) was conducted. As shown in Figure 1f, Sn 3*d*, and O 1*s* peaks are clearly shown in R‐BNP/SnO_2_ and pristine SnO_2_ (Figure S4). In addition, after incorporating R‐BNP (Figure S5), additional O 1*s* components, assigned to chemically bonded phosphate oxygen species, including bridging O (P─OH and P─O─C) and Non‐bridging O (P─O and P═O) [42], emerged in R‐BNP/SnO_2_ (Figure S6b), consistent with the successful coordination of phosphate groups to the SnO_2_ surface. The P 2*p* spectrum of pristine R‐BNP exhibits two distinct peaks at approximately 133.0 and 134.0 eV, which can be assigned to P─O/P═O and C─O─P species [43], respectively. Upon incorporation of SnO_2,_ both P 2*p* peaks shift slightly toward higher binding energies (Figure 1g), while the Sn 3*d* shifts to lower binding energies (Figure S6a). These opposite shifts indicate interfacial electronic coupling and charge redistribution between the surface‐bound R‐BNP and the SnO_2_ surface [34, 44]. The phosphate groups of R‐BNP are covalently anchored to surface hydroxyl groups through Sn─O─P linkages, a well‐established binding mode for phosphate‐functionalized molecules on metal oxide surfaces [35, 45]. Consequently, the chiral molecules are confined to the catalyst surface without incorporation into the SnO_2_ crystal lattice, consistent with the unchanged Raman, XRD, and STEM results.

**FIGURE 1:**
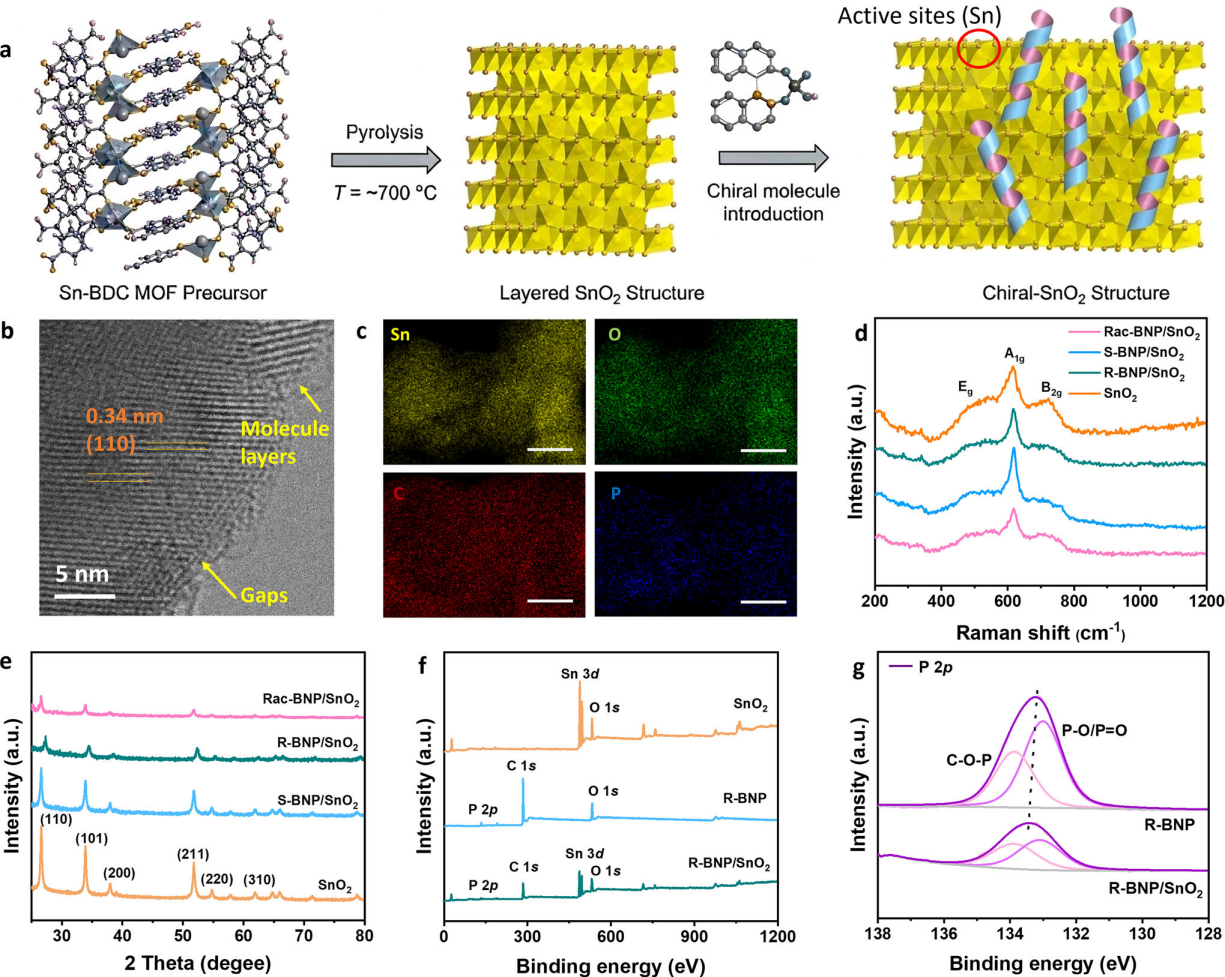
(a) Schematic for the preparation of chiral‐SnO_2_ catalyst. (b) Bright‐field (BF) scanning transmission electron microscopy (STEM) image of R‐BNP/SnO_2_ (scale bar: 5 nm). (c) EDS mapping images of R‐BNP/SnO_2_: Sn element (yellow), C element (red), O element (green), and P element (blue) (scale bar: 10 nm). (d) Raman spectra of SnO_2_, R‐BNP/SnO_2_, S‐BNP/SnO_2_, and Rac‐BNP/SnO_2_. (e) XRD patterns of SnO_2_, R‐BNP/SnO_2_, S‐BNP/SnO_2_, and Rac‐BNP/SnO_2_. (f) XPS spectra of SnO_2_, R‐BNP, and R‐BNP/SnO_2_. (g) High‐resolution XPS spectra of P 2*p* for R‐BNP and R‐BNP/SnO_2_.

### 2.2 Chiral and Racemic Properties of Various SnO_2_ Catalysts

To identify the chirality of R‐BNP/SnO_2_ and S‐BNP/SnO_2_, we employed circular dichroism (CD) spectroscopy and Kelvin probe force microscopy (KPFM) under north‐pole (N‐pole) and south‐pole (S‐pole) magnetization conditions [46]. The liquid‐phase CD spectra of the BNP molecules, including R‐BNP, S‐BNP, and Rac‐BNP, were collected (Figure 2a). R‐BNP and S‐BNP exhibited strong mirror‐image Cotton effect centered at ∼336 nm, confirming their opposite chiral configuration. In contrast, Rac‐BNP exhibits an almost CD‐silent spectrum due to the cancellation of the opposite‐enantiomeric signals. Figure 2b presents the transmitted circular dichroism (TCD) spectra of BNP‐modified SnO_2_ catalysts. Similar mirror‐symmetric CD signals are observed for R‐BNP/SnO_2_ and S‐BNP/SnO_2_, while Rac‐BNP/SnO_2_ shows negligible CD activity [47, 48]. These results indicate effective chirality transfer from chiral BNP to SnO_2_, while the increased noise at shorter wavelengths likely reflects a weaker UV signal‐to‐noise ratio and spectral broadening due to heterogeneous surface binding and partial damping of the induced chiroptical response [32]. Meanwhile, regions of interest were selected from each sample for KPFM analysis (Figure 2d,h,l). A comparison of Figures 2e,f shows that R‐BNP/SnO_2_ exhibits greater brightness under S‐pole magnetization than under N‐pole magnetization, indicating a higher contact potential difference (CPD). In contrast, S‐BNP/SnO_2_ displays a higher CPD in the N‐pole magnetization compared to the S‐pole magnetization (Figure 2i,j). These opposite magnetization‐dependent CPD responses are consistent with chirality‐dependent spin‐selective charge polarization at the catalyst interface [46]. As a control, Rac‐BNP/SnO_2_ reveals nearly identical CPD values under S‐ and N‐pole magnetization conditions (Figure 2m,n). Furthermore, the normalized potential histograms and the corresponding peak potential differences obtained from KPFM mapping provide quantitative statistical evidence of the CISS effect [49, 50]. The potential peak differences extracted from the KPFM measurements are 63 mV for R‐BNP/SnO_2_ (Figure 2g) and −40 mV for S‐BNP/SnO_2_ (Figure 2k). The opposite signs of potential shifts suggest a reversed spin‐selective charge polarization between the two enantiomeric chiral interfaces [49]. As a control, the normalized potential histogram for Rac‐BNP/SnO_2_ under both N‐ and S‐pole magnetization conditions nearly overlaps (Figure 2o), with negligible differences in peak potentials, confirming the absence of significant spin‐selective charge polarization. In summary, KPFM measurements establish a quantitative correlation between material chirality and spin‐selective interfacial charge polarization, confirming the CISS characteristics of the chiral SnO_2_.

**FIGURE 2:**
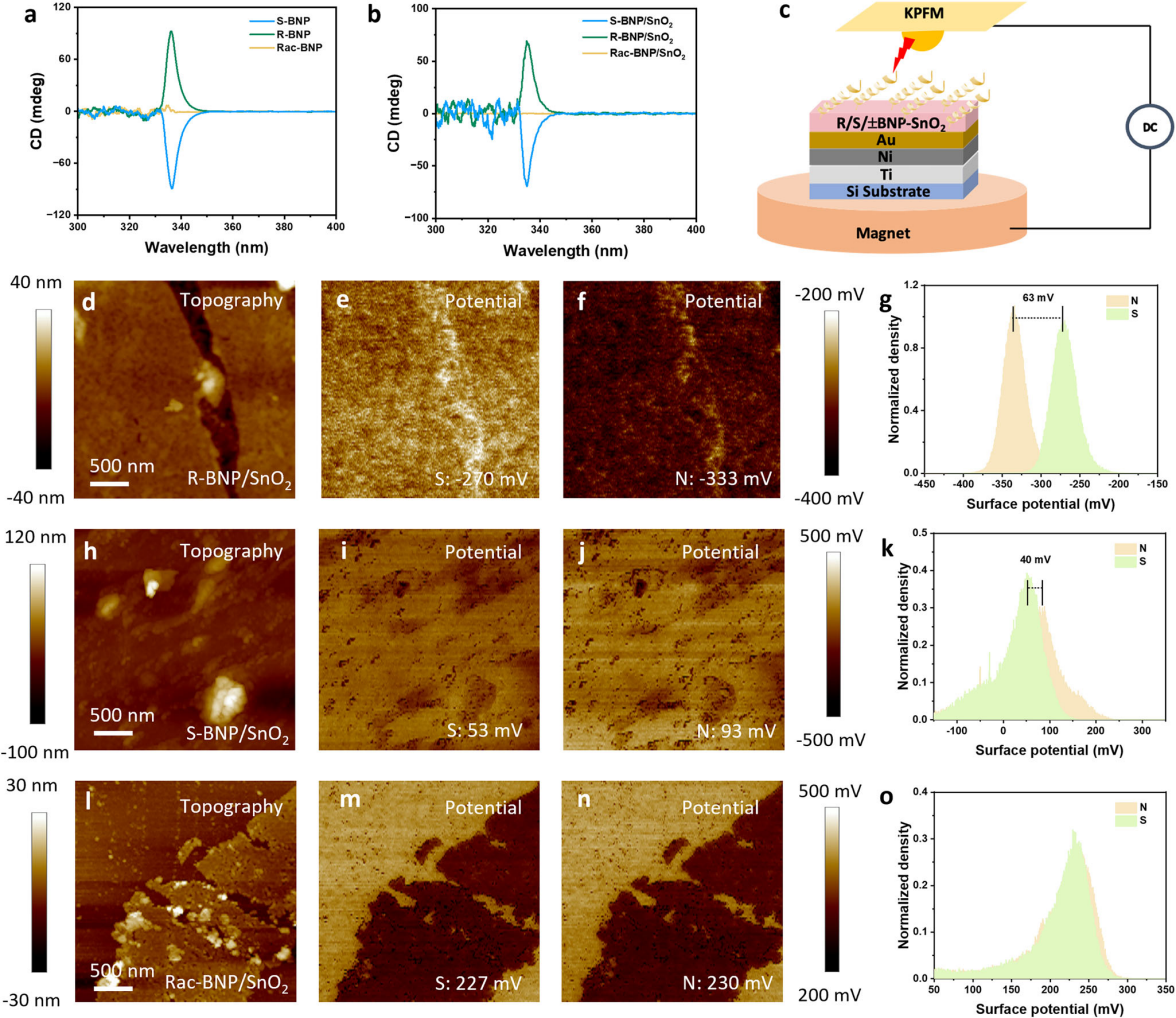
(a) Liquid‐phase CD spectra of R‐BNP, S‐BNP, and Rac‐BNP in solution. (b) Transmitted CD spectra of R‐BNP/SnO_2_, S‐BNP/SnO_2_, and Rac‐BNP/SnO_2_ samples. (c) Schematic illustration of the KPFM measurement, the KPFM study of CISS was conducted on the same location of R‐BNP, S‐BNP, and Rac‐BNP under different magnetic fields. AFM topographical images of (d) R‐BNP/SnO_2_, (h) S‐BNP/SnO_2_, and (l) Rac‐BNP/SnO_2_. Surface potential images under N‐pole and S‐pole magnetic fields, and normalized surface potential distribution histogram under different conditions of (e–g) R‐BNP/SnO_2_, (i–k) S‐BNP/SnO_2_, and (m–o) Rac‐BNP/SnO_2_ (scale bar: 500 nm).

### 2.3 Electrocatalytic CO_2_ Reduction Performance Investigation

Subsequently, the CO_2_RR activities of the chiral SnO_2_ (R‐BNP/SnO_2_ and S‐BNP/SnO_2_) were systematically compared with those of Rac‐BNP/SnO_2_ and bare SnO_2_. Linear sweep voltammetry (LSV) curves for various SnO_2_ catalysts were measured in CO_2_− and Ar‐saturated 0.5 M KHCO_3_ electrolyte (Figure 3a). Bare SnO_2_ delivered a higher current density (−6.73 mA cm^−2^ at −1.2 V) under a CO_2_ atmosphere than under an Ar atmosphere (−4.36 mA cm^−2^), demonstrating its catalytic activity toward CO_2_RR. After chiral modification, both R‐BNP/SnO_2_ and S‐BNP/SnO_2_ exhibited significantly higher current densities under CO_2_ at −1.2 V (−11.86 and −12.28 mA cm^−2^, respectively), nearly twice that of bare SnO_2_. In contrast, significantly lower current densities were observed for the chiral catalysts under an Ar atmosphere (−4.97 and −6.08 mA cm^−2^ for R‐BNP/SnO_2_ and S‐BNP/SnO_2_, respectively), confirming that enhanced current responses primarily originate from promoted CO_2_RR activity. In contrast, Rac‐BNP/SnO_2_ exhibited only a negligible difference in current density between CO_2_− and Ar‐saturated electrolytes, suggesting that nonchiral molecular coverage is not capable of enhancing CO_2_RR activity [51]. To further support this observation, Figure 3b summarizes the faradaic efficiency (FE) of carbon‐based products and competing HER (Tables S1–S4). Notably, both chiral catalysts exhibit strong capabilities to suppress HER. At −1.2 V, the H_2_ FEs for R‐BNP/SnO_2_ and S‐BNP/SnO_2_ are only 22.8% and 32.7%, respectively, which are substantially lower than those observed for bare SnO_2_ (52.4%) and Rac‐BNP/SnO_2_ (56.5%). According to previous studies, HER is initiated by the formation of adsorbed hydrogen (H*) on the catalytic surface [52, 53]. Subsequently, H_2_ is then generated either through the Tafel pathway, involving chemical recombination of two adjacent H* species, or through the Heyrovsky pathway, in which surface‐bound H* reacts electrochemically with a solvated proton and electron [52, 53]. These elementary steps involve electron transfer to adsorbed intermediates, like H*, and therefore may be influenced by spin‐dependent electron‐transfer processes. At a spin‐polarized electrocatalytic interface, the CISS effect may suppress HER by preferentially aligning electron spins in parallel, thereby creating a spin‐related kinetic barrier for the formation of the singlet‐state H_2_ molecule. Meanwhile, spin polarization may lower the kinetic barrier for CO_2_RR relative to HER, thereby increasing the fraction of electrons utilized for CO_2_RR. As a result, suppressing HER favors the competing CO_2_RR pathways and promotes efficient electron utilization toward carbon‐based products.

**FIGURE 3:**
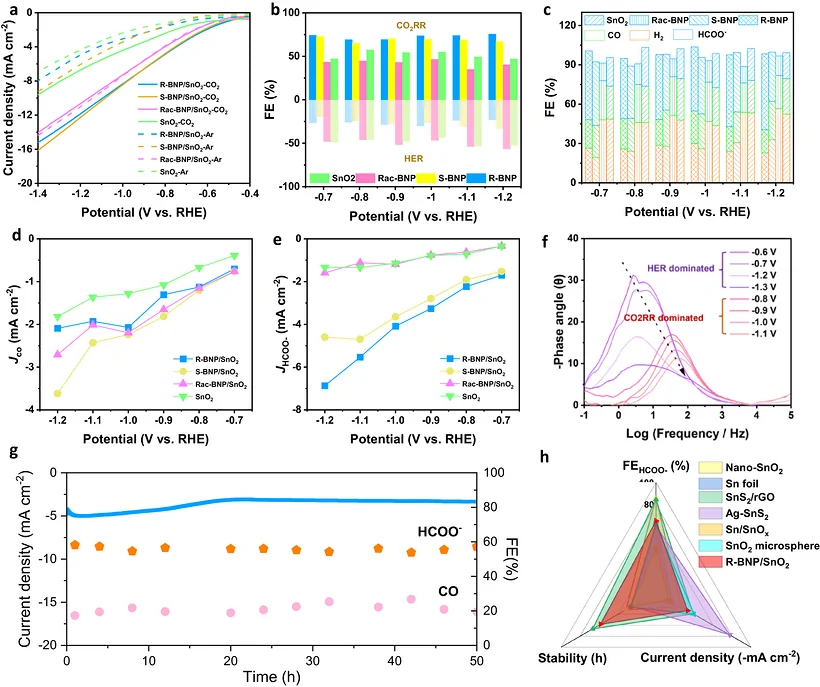
LSV curves of SnO_2_, R‐BNP/SnO_2_, S‐BNP/SnO_2_, and Rac‐BNP/SnO_2_ catalysts under an Ar atmosphere or a CO_2_ atmosphere. (b) FEs of carbon product (HCOO^−^+CO) and hydrogen product (H_2_) over SnO_2_, R‐BNP/SnO_2_, S‐BNP/SnO_2_, and Rac‐BNP/SnO_2_. (c) FEs of HCOO^−^, CO, and H_2_ over SnO_2_, R‐BNP/SnO_2_, S‐BNP/SnO_2_, and Rac‐BNP/SnO_2_. Partial current density of CO (d) and HCOO^−^ (e) over SnO_2_, R‐BNP/SnO_2_, S‐BNP/SnO_2_, and Rac‐BNP/SnO_2_. (f) In situ EIS measurements of R‐BNP/SnO_2_ and phase angle versus frequency plots (from −0.6 to −1.3 V vs. RHE). (g) Stability test of R‐BNP/SnO_2_ under −1.2 V versus RHE over 50 h. (h) Performance comparison with previously reported Sn‐based catalysts.

Under these conditions, CO and formate were identified as the primary CO_2_RR products (Figure 3c). The product selectivity results reveal a pronounced enhancement in formate production over the chiral catalysts. At −1.2 V, R‐BNP/SnO_2_ achieves FEs of 57.9% for formate and 17.6% for CO, while S‐BNP/SnO_2_ delivers 37.5% formate and 29.4% CO, respectively. In comparison, bare SnO_2_ exhibits substantially lower formate selectivity, producing 19.9% formate and 27.0% CO, whereas Rac‐BNP/SnO_2_ shows similar behavior with 14.9% formate and 25.4% CO. Notably, bare SnO_2_ displays a formate‐to‐CO ratio of approximately 1:1.4, indicating a slight preference for CO formation on the unmodified surface. To distinguish the influence of chirality from general molecule modification effects, the racemic catalyst showed only a minor change in selectivity relative to bare SnO_2_, suggesting that molecular coverage or electronic perturbations alone do not significantly alter the reaction pathway. In contrast, enantiopure chiral modification dramatically shifts the formate‐to‐CO ratio from ∼1:1.4 to ∼3.3:1, converting the product distribution from CO‐dominant to formate‐dominant.

Correlated with selectivity results, the partial current of CO (*J_CO_*) is slightly increased for chiral SnO_2_ compared to bare SnO_2_, especially at higher potential, indicating the CISS effect could suppress HER (Figure 3d). At the same time, both chiral catalysts, R‐BNP/SnO_2_ and S‐BNP/SnO_2_, exhibit substantially enhanced formate partial current densities (*J*_HCOO‐_) compared with bare SnO_2_ and Rac‐BNP/SnO_2_ across the investigated potential range (Figure 3e). In particular, R‐BNP/SnO_2_ delivers the highest formate current density at more negative potentials, indicating that chiral modification significantly promotes the formate production pathway. In contrast, bare SnO_2_ has the lowest *J*_HCOO‐_ values, consistent with its poor formate selectivity. Rac‐BNP shows similar performance as bare SnO_2_, further suggesting that chirality rather than simple molecular modification is responsible for the improved formate formation.

To identify the active sites for CO_2_RR, we first evaluated the intrinsic activity of the chiral molecules. As shown in Figure S7, pure R‐BNP and S‐BNP deposited on an inert carbon substrate exhibited negligible CO_2_RR activity and extremely low current densities, indicating that the chiral molecules themselves do not serve as the primary catalytic center. We further prepared SnO_2_ modified with the corresponding racemic structural analog (Rac‐BNP/SnO_2_) as a control. As shown in Figure 3a–c, both R‐BNP/SnO_2_ and S‐BNP/SnO_2_ deliver significantly higher FEs and current densities than Rac‐BNP/SnO_2_ under identical conditions. Since the chiral and racemic modifiers possess nearly identical chemical structures but differ in their ability to induce spin polarization, these results demonstrate that the enhanced CO_2_RR performance originates from the CISS effect rather than from the molecular modification itself.

To distinguish the contribution of the CISS effect from conventional interfacial factors, such as catalyst wettability and local microenvironment, we next performed water contact angle measurements. As shown in Figure S8a, the pristine CFP substrate exhibits a contact angle of 85.647°, indicating a relatively hydrophobic surface. In contrast, after catalyst loading, bare SnO_2_ and all BNP‐modified SnO_2_ electrodes exhibited instantaneous spreading of the water droplet, precluding reliable static contact angle determination (Figure S8b). This behavior can be explained by the intrinsically hydrophilic nature of SnO_2_, its abundant surface hydroxyl groups, and the porous architecture of the catalyst layer [54]. These results indicate that BNP modification does not produce measurable changes in the macroscopic wettability of the electrode.

To further investigate the influence of BNP coverage, we measured the surface wettability with different BNP loadings. All samples exhibited instantaneous water spreading, indicating no detectable difference in macroscopic hydrophilicity (Figure S8b). Nevertheless, the CO_2_RR performance exhibited a volcano‐shaped dependence on BNP coverage (Figure S9), with an optimal R‐BNP coverage (40%). At lower coverage (20%), the density of chiral molecules is likely insufficient to generate an effective CISS‐mediated interfacial modulation. In contrast, excessive R‐BNP loading (80%) may partially block exposed Sn active sites and introduce steric hindrance for CO_2_ adsorption and intermediate conversion, resulting in decreasing catalytic activity (Figure S9). Therefore, the volcano‐shaped dependence of CO_2_RR performance on BNP coverage is more reasonably attributed to the balance between the effective chiral interfacial regulation and preservation of accessible catalytic sites, rather than to changes in macroscopic wettability.

In situ electrochemical impedance spectroscopy (in situ EIS) was used to investigate interfacial charge‐transfer behavior and reaction kinetics during CO_2_RR [55]. The equivalent circuit model was used to fit the EIS measurements (Figure S10). As shown in Figure 3f, the low‐frequency response is associated with interfacial electrochemical processes at the electrode‐electrolyte interface, including charge transfer and surface reaction dynamics related to HER and CO_2_RR [55]. For R‐BNP/SnO_2_, at relatively mild cathodic potential (−0.6 to −0.7 V vs. RHE), the spectra exhibit larger phase angles centered at lower frequency, consistent with a HER‐dominated interfacial process [55]. As the applied potential becomes more negative, the characteristic relaxation feature progressively shifts toward higher frequency and lower phase angles. Notably, between −0.8 and −1.1 V versus RHE, a distinct evolution in the phase response is observed, which correlates well with the onset and increasing contribution of CO_2_RR. In contrast, such pronounced spectral evolution is absent for Rac‐BNP/SnO_2_ and bare SnO_2_ (Figure S11 and Table S5), suggesting that the chiral interface modified interfacial reaction kinetics and promotes CO_2_RR relative to the HER, consistent with the FE and partial current density results.

To investigate whether the catalyst's active surface area after organic molecule integration could lead to significant differences in CO_2_RR performance, electrochemically active surface area (ECSA) tests were conducted (Figure S12). As shown in Figure S13, all SnO_2_ variants exhibit comparable electrical double‐layer capacitance (C*_dl_*) values, indicating that the introduction of organic molecules does not significantly alter the intrinsic catalytic activity. Lastly, to rule out that the enhanced CO_2_RR performance originates from the decomposition of surface‐adsorbed BNP, we examined the long‐term stability of R‐BNP/SnO_2_. After 50 h of electrolysis under CO_2_, both the current density and FEs for CO and HCOO^−^ show minimal changes, indicating that the catalyst preserves its structural integrity during prolonged operation (Figure 3g). The P 2*p* peak associated with BNP is clearly preserved in the post‐reaction XPS spectra (Figure S14), and the transmitted CD spectra after electrolysis maintain a symmetric profile (Figure S15), indicating that the electrodes still maintain their chirality under the reaction conditions. To further evaluate the structural stability of the electrode, electrolyte samples collected before and after electrolysis were analyzed by ^1^H NMR spectroscopy (Figure S16). No detectable signals corresponding to dissolved BNP molecules were observed after electrolysis, suggesting negligible catalyst dissolution or film delamination during electrolysis. Collectively, these results demonstrate that chiral SnO_2_ achieves a well‐balanced performance profile, combining high formate selectivity with superior current density and long‐term stability, competitive with state‐of‐the‐art Sn‐based catalysts for CO_2_ reduction (Figure 3h).

### 2.4 Mechanistic Study of the CISS Effect on Intermediate Generation and Reaction Pathway

To elucidate the role of CISS in governing intermediate formation and reaction pathways, in situ attenuated total reflection surface‐enhanced infrared (ATR–SEIRAS) spectroscopy was performed on SnO_2_, R‐BNP/SnO_2_, and Rac‐BNP/SnO_2_. Under CO_2_‐saturated electrolysis conditions, all catalysts exhibit a broad peak centered at ∼1655 cm^−1^, which is attributed to adsorbed H_2_O species (H_2_O*), indicating that different catalysts possess broadly comparable interfacial hydration environments [16].

Distinct differences emerge in the intermediate regions upon chiral modification (Figures 4a–c and S17,S18). For R‐BNP/SnO_2_, two characteristic peaks located at 1445 and 1365 cm^−1^ are observed, whereas bare SnO_2_ shows a dominant feature at ∼1365. The 1445 cm^−1^ band is assigned to O‐bound *OCHO species [56], which is widely recognized as a key intermediate in the formate production pathway (Figures 4b and S19). Notably, the *OCHO signal progressively intensifies as the cathodic potential increases, suggesting enhanced stabilization and accumulation of formate‐associated intermediates on the chiral interface. By contrast, the ∼1365 cm^−1^ band can be assigned to carboxylate‐related C─O stretching vibrations associated with *COOH or COO‐derived intermediates. On bare SnO_2_, the ∼1365 cm^−1^ feature increases markedly with increasing potential (Figure 4a), consistent with preferential stabilization of *COOH‐related intermediates and the observed CO selective behavior [16]. Meanwhile, only weak *OCHO‐related features are detected on bare SnO_2_, implying limited stabilization of the formate pathway.

**FIGURE 4:**
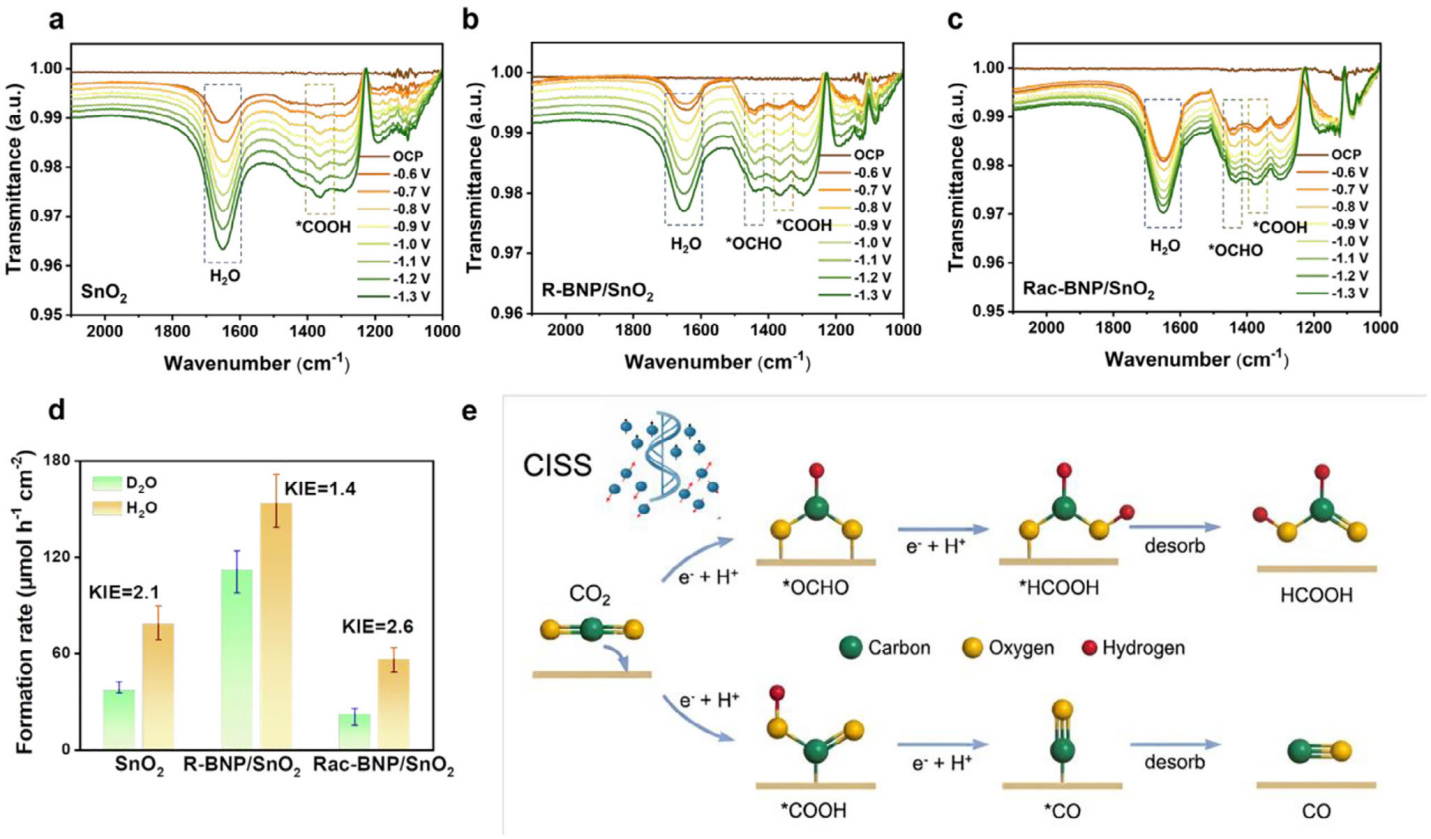
In situ ATR–SEIRAS spectra for (a) SnO_2_, (b) R‐BNP/SnO_2_, and (c) Rac‐BNP/SnO_2_. (d) CO and HCOO^−^ formation rates, and KIE values of SnO_2_, R‐BNP/SnO_2_, and Rac‐BNP/SnO_2_. (e) Schematic illustration of spin‐polarized electrons modulating possible relative energetics of competing hydrogenation pathways through interactions with potential spin‐active intermediates.

Interestingly, Rac‐BNP/SnO_2_ (Figure 4c) exhibits features associated with both *COOH and *OCHO intermediates; however, their peak intensities show only minimal changes with increasing potential (Figure S19). As shown in Figure S18, the characteristic intermediate bands on R‐BNP/SnO_2_ gradually decrease and disappear during the reverse scan (from −1.2 to −0.6 V vs. RHE), further confirming that these bands correspond to electrochemically generated surface intermediates rather than irreversible changes of the catalyst surface. For comparison, only weak spectral evolution is observed for Rac‐BNP/SnO_2_ over the same potential range, indicating substantially lower accumulation of these intermediates under identical experimental conditions. These observations indicate that the racemic BNP modifier might stabilize or influence interfacial adsorption behavior but does not effectively direct the CO_2_RR pathway. In addition, a relatively strong H_2_O* signal is observed even at low potentials (i.e., −0.7 V) and further increases with applied bias, suggesting substantial HER activity even under a small overpotential. In contrast, the enantiopure chiral interfaces simultaneously promote *OCHO stabilization while suppressing HER, thereby shifting the reaction pathway toward formate production. Collectively, these ATR–SEIRAS results suggest that the chiral interface modulates the stabilization and evolution of CO_2_RR intermediates, favoring the *OCHO‐mediated formate pathway. The spectroscopic observations are in excellent agreement with the partial current densities (Figure 3e) and product selectivity measurements (Figure 3b). To further elucidate the role of the interfacial water structure, the OH stretching region was deconvoluted into strongly and weakly hydrogen‐bonded water components (Figures S20–S22). The strong/weak hydrogen‐bond ratio was subsequently used to evaluate the evolution of the interfacial hydrogen‐bonding network under CO_2_RR conditions (Figure S23). Specifically, the band centered at ∼3250 cm^−1^ is assigned to strong hydrogen‐bonded water, whereas the feature at ∼3450 cm^−1^ corresponds to weak hydrogen‐bonded water [57, 58]. Bare SnO_2_ and Rac‐BNP/SnO_2_ exhibit a pronounced increase in the ratio of strong/weak hydrogen‐bond ratio with increasing cathodic potential, indicating progressive reorganization of the interfacial water network toward a more ordered and strongly hydrogen‐bonded structure. Such potential‐dependent strengthening of the hydrogen‐bond network is commonly associated with enhanced interfacial proton transfer and accelerated water dissociation kinetics, both of which favor the HER process [57, 58]. In contrast, R‐BNP/SnO_2_ maintains a substantially lower strong/weak hydrogen‐bond ratio across the entire potential range with only minimal potential‐dependent variation, suggesting that the chiral interface suppresses electric‐field‐driven reconstruction of interfacial water. The weakened hydrogen‐bond connectivity and restricted water reorientation at the spin‐polarized chiral interface offer a molecular‐level explanation for the effective suppression of HER by the CISS effect.

Though previous CISS studies have provided indirect evidence that CISS can suppress HER, this conclusion was primarily inferred from enhanced FEs for CO_2_RR products accompanied by decreased selectivity toward HER [26, 53]. However, such observations remain largely circumstantial, as they do not directly reveal how spin polarization influences the adsorption and transformation of surface intermediates. In this context, the present work provides experimental evidence supporting that CISS‐induced spin polarization modulates the competition between CO_2_RR and HER.

### 2.5 Mechanism Insight From KIE Measurements

To further probe the impact of the CISS effect on reaction kinetics, kinetic isotope effect (KIE) measurements (Figure 4d) were conducted [59]. Electrolysis was performed in both H_2_O and D_2_O electrolytes at −1.2 V vs. RHE. The KIE values were determined from the formation rates of carbon‐containing products (CO and HCOO^−^), quantified by GC and NMR after 5 min of electrolysis. Due to the higher dissociation energy of the O─D bond compared to the O─H bond, all catalysts exhibit slower reaction kinetics in D_2_O. When the KIE value is greater than 1, it suggests that proton transfer is the rate‐determining step (RDS) [59]. However, when the KIE value is close to 1, it suggests that proton transfer is not involved in the RDS. Thus, the KIE value could serve as a descriptor to evaluate the RDS in CO_2_RR [59]. For bare SnO_2_ and Rac‐BNP/SnO_2_ catalysts, the KIE values are calculated to be 2.1 and 2.6, respectively, indicating that proton transfer was the RDS in CO_2_RR (Figure 4d). In contrast, R‐BNP/SnO_2_ shows a significantly lower KIE value of 1.4. The reduction in KIE implies that the introduction of chiral molecules accelerates proton‐coupled electron‐transfer steps, thereby lowering the barrier associated with proton transfer. As a result, R‐BNP/SnO_2_ suppresses HER and selectively enhances CO_2_RR.

Together with the ATR–SEIRAS results, these findings suggest that CISS not only modulates intermediate stabilization but also influences reaction kinetics. Although the two‐electron reduction pathways to formate and CO are thermodynamically similar, they proceed through distinct adsorption configurations [60]: O‐bound *OCHO for formate and C‐bound *COOH for CO (Figure 4e). On bare SnO_2_, the catalyst surface preferentially stabilizes the *COOH species, which subsequently undergoes proton‐coupled electron transfer and dehydroxylation to generate adsorbed *CO, ultimately leading to CO evolution [61]. In contrast, R‐BNP/SnO_2_ interface selectively promotes the stabilization and accumulation of *OCHO, thereby redirecting the reaction pathway toward formate production [25]. Therefore, spin‐polarized electrons may influence competing CO_2_ hydrogenation pathways by modulating spin‐dependent electronic structures of reaction intermediates and altering relative energy barriers of elementary reaction steps. This change in intermediate preference reshapes the reaction landscape and enables selective control over competing pathways that are otherwise close in thermodynamic energy.

## 3 Conclusion

In summary, we demonstrate a transformative approach for regulating CO_2_RR through the CISS effect. By integrating chiral BNP molecular interfaces with SnO_2_, the reaction pathway is markedly redirected from CO‐dominant production toward formate‐dominant conversion. Unlike conventional approaches based on structural or electronic tuning, the chiral interface introduces a spin‐selective interfacial effect that fundamentally alters the stabilization of intermediates and reaction kinetics. In situ ATR‐SEIRAS measurements provide direct mechanistic evidence that the chiral interface selectively promotes the adsorption, stabilization, and accumulation of the O‐bound *OCHO intermediate. In addition, chiral SnO_2_ maintains a substantially lower strong/weak hydrogen‐bond ratio, suggesting that the chiral interface suppresses electric‐field‐driven reconstruction of interfacial water, therefore suppressing competitive HER. This selective modulation of competing intermediates effectively reshapes the interfacial reaction landscape and enables control over reaction pathways with close thermodynamic energetics.

## Author Contributions

F.H., J.G., and Y.Y. conceived the idea. F.H. performed experiments. X.Y and X.P. performed the TEM and EDS experiments. F.H. and J.G. wrote and modified the manuscript. Y.Y. gave valuable suggestions throughout the study. All authors discussed the results and commented on the manuscript.

## Conflicts of Interest

The authors declare no conflicts of interest.

## Supporting information



The authors have cited additional references in the Supporting Information [62].
**Supporting File**: anie74319‐sup‐0001‐SuppMat.docx.

## Data Availability

The data are available from the corresponding authors upon reasonable request.
