# Supplementary material for: Spin‐Polarized Interfaces Redirect CO2 Reduction From CO to Formate
Source: Angew Chem Int Ed Engl. 2026 Aug 20;65(42):e1729522. doi: 10.1002/anie.1729522 (PMC13653629; doi:10.1002/anie.1729522)
Supplement: Supplementary file 1 [file ANIE-65-e1729522-s001.docx]

Spin-Polarized Interfaces Redirect CO_2_ Reduction from CO to Formate

Fan He^1*^, Xingxu Yan^2^, Xiaoqing Pan^2,3^, Yong Yan^1^, Jing Gu^1*^

^1^ Department of Chemistry and Biochemistry, San Diego State University, San Diego, CA 92182, USA

^2^ Department of Materials Science and Engineering, University of California, Irvine, CA 92697, USA

^3^ Department of Physics and Astronomy, University of California, Irvine, Irvine, CA 92697, USA

^*^Corresponding author. Email: fhe@sdsu.edu; jgu@sdsu.edu

**Experimental sections**

**Chemicals**

All chemicals were purchased and used as received unless otherwise stated. Tin (II) sulfate (SnSO_4_), terephthalic acid (1,4-BDC), ammonium hydroxide (NH_3_·H_2_O), deuterium oxide (D_2_O), and N, N-dimethylformamide (DMF), R-1,1′-Bi-2-naphthyl-2,2′-diyl hydrogen phosphate (R-BNP), S-1,1′-Bi-2-naphthyl-2,2′-diyl hydrogen phosphate (S-BNP), and Racemic-1,1′-Bi-2-naphthyl-2,2′-diyl hydrogen phosphate (Rac-BNP) were all purchased from Fisher Scientific. Deuterium oxide (99.9 atom %D) was purchased from Oakwood Chemical.

**Synthesis of SnO_2_**

Typically, 1,4-BDC (12 mmol) and NH_3_·H_2_O (24 mmol) were dissolved in the mixed solvent of 50 mL DMF and deionized (DI) water to form a clear solution. Further, SnSO_4_ (5 mmol) is dispersed in 20 mL of DI water and then slowly added to the 1,4-BDC solution under vigorous stirring. After thorough mixing for about 2 h, the resulting precipitate was suspended in water for 1 h. Finally, the product was collected by centrifugation, washed with DMF and DI water three times, and dried under vacuum at 70 °C for 12 h. Pure SnO_2_ was obtained after annealing Sn-BDC at 500 °C with a heating rate of 1 °C min^-1^ in the air for 3h.

**Synthesis of R-BNP/SnO_2_**

2 mg of R-BNP and 5 mg of as-prepared SnO_2_ were ultrasonically dispersed in 500 μL of Nafion ethanol solution (containing 50 μL of 0.5 wt% Nafion and 450 μL of ethanol) for 1 h. Further, the mixture was stirred overnight to form uniformly dispersed inks, respectively. Afterward, 100 μL drops of R-BNP/SnO_2_ ink were evenly dispersed on the carbon fiber paper substrate.

**Synthesis of S-BNP/SnO_2_**

2 mg of S-BNP and 5 mg of as-prepared SnO_2_ were ultrasonically dispersed in 500 μL of Nafion ethanol solution (containing 50 μL of 0.5 wt% Nafion and 450 μL of ethanol) for 1 h. Further, the mixture was stirred overnight to form uniformly dispersed inks, respectively. Afterward, 100 μL drops of S-BNP/SnO_2_ ink were evenly dispersed on carbon fiber paper substrate.

**Synthesis of Racemic-BNP/SnO_2_ (Rac-BNP/SnO_2_)**

2 mg of Racemic-BNP and 5 mg of as-prepared SnO_2_ were ultrasonically dispersed into 500 μL Nafion ethanol solution (containing 50 μL of 0.5 wt% Nafion and 450 μL of ethanol) for 1 h. Further, the mixture was stirred overnight to form uniformly dispersed inks, respectively. Afterward, 100 μL drops of Rac-BNP/SnO_2_ ink were evenly dispersed on carbon fiber paper substrate.

**Characterizations**

**Scanning electron microscopy**: The morphologies of as-prepared samples were obtained by scanning electron microscopy (SEM) using the FEI Quanta 450 FEG SEM at 20 KeV.

**Scanning transmission electron microscopy**: Scanning transmission electron microscopy (STEM) was performed using JEOL JEM-ARM300F Grand ARM S/TEM, and bright-field (BF)-STEM images were acquired at a convergence semiangle of 22 mrad and a BF detector collection semiangle of 38 mrad, respectively. Energy dispersive X-ray spectroscopy mapping results were captured using the dual high-speed and high-solid-angle silicon drift detectors in the same microscope.

High spatial resolution STEM-EELS experiments were performed by Nion UltraSTEM 200 at UC Irvine, equipped with a C3/C5 corrector and a high-energy resolution monochromated EELS system (HERMES). The instrument was operated at 60kV with a convergence semi-angle of 38 mrad and a beam current of ~10 pA. For STEM imaging, the inner and outer collection semi-angles of the high-angle and medium-angle annular dark-field (HAADF) detector were 95 and 210 mrad, respectively, while the collection semi-angle of the bright-field (BF) detector was 49 mrad.

**X-ray powder diffraction**: The crystal structures of as-prepared samples were analyzed by X-ray powder diffraction (XRD) recorded on PROTO AXRD benchtop powder X-ray diffractometer at 40 kV and 30 mA using Ni-filtered Cu Kα radiation (λ = 1.5406 Å) over a 2-theta range of 5-80 degrees.

**X-ray photoelectron spectroscopy**: The chemical structures of as-prepared samples were measured by X-ray photoelectron spectroscopy (XPS, PHI 5600) with Al *K*_α_ radiation. All XPS spectra were corrected by calibrating the peaks to the major adventitious C-C peak at 284.8 eV ^62^.

**Fourier transform infrared Spectroscopy**: Fourier transform infrared (FTIR) spectra were obtained using a PERKIN ELMER FTIR spectrometer.

**Raman Spectroscopy**: Raman spectra were recorded using a Thermo Scientific DXR Raman microscope with an Ar-ion laser at 532 nm.

**Electrochemical measurements**

All electrochemical measurements were carried out at 25 °C using an electrochemical workstation (CHI 660E). A customized gas-tight H-type glass cell with a Nafion 117 membrane was employed to separate cell compartments and ensure gas tightness during the experiments. In a typical three-electrode system, a platinum wire and an Ag/AgCl electrode were used as the counter and reference electrode, respectively. The working electrode was a 1 × 1 cm^2^ carbon paper with a catalyst loading amount of 1.0 mg cm^-2^. Before measurements, the cell was sparged with CO_2_ at 20 sccm for 30 min. Then, the polarization curves were recorded over the potential range of -0.2 V to -1.4 V (vs. RHE) at a scan rate of 5 mV s^-1^. The polarization curves were further collected after cyclic voltammograms (CV) activation over the same potential windows. The CV curves used for the ECSA calculations were scanned from 0 V to -0.1 V (vs. RHE). The EIS measurements were carried out at -0.5 V (vs. RHE) with a 100 mV amplitude over a frequency range from 100 kHz to 100 mHz. In this work, online GC and offline ^1^H NMR measurements were used to analyze the gaseous and liquid products, respectively, and their FEs at different applied potentials. All potentials measured against Ag/AgCl (*E*_Ag/AgCl_) were converted to the RHE (*E*_RHE_) scale in this work using *E*_RHE_ = *E*_Ag/AgCl_ × 0.197 V + 0.0591 × pH. The electrolyte in the anodic compartment was stirred at 2,000 rpm during electrolysis. All measured potentials were manually compensated unless otherwise stated.

**Electrochemically active surface area (ECSA) calculation**

The electrochemically active surface area (ECSA) of an electrocatalytic material can be estimated by measuring the double-layer capacitance (C*_dl_*) of the electrode-electrolyte interface. The C*_dl_* value can be measured by conducting CV in a potential window where no Faradaic processes occur. In this potential region, any measured current can be attributed to the non-Faradaic process of charging the electrochemical double layer. The charging current *j* measured during CV is linearly related to the scan rate *v,* with a slope equal to C*_dl_*. For all the electrocatalysts, the CV potential window was 0 V to -0.1 V versus RHE in a CO_2_-saturated 0.5 M KHCO_3_ electrolyte.

**Products analysis**

Controlled potentiostaic electrolysis was carried out at each potential for 20 min. The composition and distribution of gas products were analyzed by gas chromatography (SHIMADZU, GC-2010) equipped with a BID detector at 10 min intervals, and the liquid products were detected by ^1^H nuclear magnetic resonance (^1^H NMR).

The CO/H_2_ Faradaic efficiency of gas products was calculated by the following equation:

$$FE=\frac{N*F\left( \frac{C}{mol} \right)*V\left( sccm \right)*c\left( ppm \right)*{10}^{-6}*{10}^{-6}\left( \frac{m^{3}}{mL} \right)*101325\left( \frac{N}{m^{2}} \right)}{8.314\left( N*\frac{m}{mol}*K \right)*298.15K*I_{total}\left( \frac{C}{s} \right)*60\left( \frac{s}{min} \right)}$$

The formate Faradaic efficiency of liquid products was calculated by the following

$$FE=\frac{generated formate \left( \mathrm{mol} \right)\times2\times96485}{the total amount of charge passed (C)}$$

For the stability test, a continuous three-electrode H-type cell was employed to continuously produce CO and formate at a constant current.

**Circular dichroism (CD) and Transmitted circular dichroism (TCD)**

The CD spectra of the samples were obtained using a JASCO J-815 spectrometer. During the measurements, the system was purged with N_2_ gas. The CD and TCD measurements require transparent or otherwise optically well-controlled samples, since the measurements depend on clean, polarization-resolved light transmission or reflection.

To fabricate the samples, R/S/Rac-BNP solutions were prepared by dissolving 0.5 mmol of R/S/Rac-BNP in 100 mL of ethanol. Then, 2 mL from the prepared solution was transferred into a cuvette. The measurement conditions for all spectra were done at a scan range of 210 to 400 nm. Three scans were collected and averaged for each measurement, with a bandwidth of 1.00 nm and a scan rate of 100 nm min^-1^. The quartz cuvette used had an optical pathway of 1 cm.

20 mg of R-BNP/SnO_2_, S-BNP/SnO_2_, and Rac-BNP/SnO_2_ powders were dispersed in 10 mL of ethanol. The resulting suspensions were transferred into a cuvette for TCD measurements. The measurement conditions for all spectra were done at a scan range of 210 to 400 nm. Three scans were collected and averaged for each measurement, with a bandwidth of 1.00 nm and a scan rate of 100 nm min^-1^.

**Kelvin probe force microscopy (KPFM) sample preparation**

10 nm of Ti, 80 nm of Ni, and 10 nm of Au were sequentially deposited onto an *n*-type silicon wafer (1 cm × 1 cm) using electron-beam deposition in a vacuum chamber at a pressure of 10^−7^ torr. The metal-deposited substrates were cleaned in boiling isopropanol for 10 mins. The precursor solution was prepared by dissolving the 5 mg S-BNP/SnO_2_, R-BNP/SnO_2_, or Rac-BNP/SnO_2_ sample in N, N-Dimethylformamide (DMF), separately, then a 100 μL solution was spin-coated onto the substrate at 4000 rpm for 30 s, followed by annealing at 80 °C for 10 mins.

**Kelvin probe force microscopy (KPFM) Testing**

The KPFM used in this experiment is a home-built system based on the Bruker Dimension Icon atomic force microscope (AFM), specifically designed to measure surface potential and assess sample work function. KPFM operates by detecting the contact potential difference (CPD) between the probe and the sample, achieved by continuously applying a DC voltage to nullify the Coulomb force. In this setup, potential data were acquired using external high-performance data-separation, filtering, and feedback equipment, and a low-noise lock-in amplifier (Zurich Instruments HF2LI). This approach enables simultaneous acquisition of both sample topography and surface potential information, yielding an electrical resolution exceeding 10 mV and a spatial resolution better than 30 nm. A conductive probe (PPP-EFM-20) was used, featuring a Pt-Ir coating across the entire front surface to ensure excellent electrical performance.

The sample was magnetized for approximately 1 h, after which KPFM measurements were performed under three conditions: no magnetic field, North-pole magnetization, and South-pole magnetization. To minimize the influence of sample variation, surface potential data were recorded from the same microscopic area under each condition.

**In-situ Attenuated Total Reflectance Surface-Enhanced Infrared Absorption Spectroscopy (ATR-SEIRAS)**

In-situ ATR-SEIRAS measurements were conducted on a vacuum Fourier-transform infrared spectrometer (Bruker, VERTEX 70 V). The spectra were all obtained in a custom-made three-electrode cell. The catalyst ink was deposited on a gold-coated silicon hemisphere (resistance <5 Ω) as the working electrode. The platinum plate and Ag/AgCl electrode were used as the counter electrode and reference electrode, respectively. 0.5 M KHCO_3_ purged with CO_2_ was the electrolyte (15 sccm CO_2_ flow for 1 h), and the tested pH of the electrolyte is 8.5. The chronoamperometric tests from −0.4 to −1.4 V vs. RHE were carried out on an electrochemical workstation (Gamry Instruments, INTERFACE 1010E 36066), accompanied by the spectrum collection. All spectra were collected after background subtraction. Three scans for each spectrum at each potential, and the electrolyte has been refreshed and re-saturated with CO_2_ for each sample.

**Figure S1**: Proposed structural diagram of chiral-SnO_2_ catalysts for CO_2_RR.

**Figure S2.** SEM images of (a) SnO_2_ and (b) R-BNP/SNO_2_.

**Figure S3.** BF STEM images and EDX mapping of SnO_2_, which show the lattice fringe of SnO_2_ and spatial distribution of Sn and O elements.

**Figure S4.** High-resolution XPS spectra of (a) O 1*s* and (b) Sn 3*d* regions for SnO_2_.

**Figure S5.** High-resolution XPS spectra of (a) O 1*s*, (b) C 1*s*, and (c) P 2*p* regions for the R-BNP sample.

**Figure S6.** High-resolution XPS spectra of (a) Sn 3*d*, (b) O 1*s*, (c) C 1*s*, and (d) P 2*p* for SnO_2_, R-BNP, and R-BNP/SnO_2_.

In addition, after incorporating R-BNP, additional O 1*s* components, assigned to chemically bonded phosphate oxygen species, including bridging O (P-OH and P-O-C) and Non-bridging O (P-O and P=O), emerged in R-BNP/SnO_2_ (**Figure S6b**), consistent with the successful coordination of phosphate groups to the SnO_2_ surface and confirming the presence of BNP. The P 2*p* spectrum of pristine R-BNP exhibits two distinct peaks at approximately 133.0 eV and 134.0 eV, which can be assigned to P-O/P=O and C-O-P species, respectively. Upon incorporation of SnO_2,_ these peaks shift slightly to higher binding energies (**Figure S6d**). This positive shift in the P 2*p* peaks, combined with the negative shift of the Sn 3*d* peaks (**Figure S6a**), suggests interfacial electronic coupling and electron redistribution between R-BNP and the SnO_2_ matrix.

**Figure S7**. LSV curves of SnO_2_, R-BNP/SnO_2_, S-BNP/SnO_2_, R-BNP, and S-BNP catalysts under a CO_2_ atmosphere.

**Figure S8**. Contact angle measurement of (a) carbon fiber paper (CFP) substrate and (b) catalysts on the CFP (SnO_2_, R-BNP/SnO_2_, S-BNP/SnO_2_, Rac-BNP/SnO_2_, and R-BNP/SnO_2_ with different coverage degree of chiral layer).

As shown in Figure S8a, the pristine CFP exhibits a contact angle of 85.647^°^, indicating a relatively hydrophobic surface. In contrast, after catalyst deposition, bare SnO_2_ and all BNP-modified SnO_2_ electrodes exhibited instantaneous spreading of the water droplet, precluding reliable static contact angle measurements. This phenomenon can be explained by the intrinsically hydrophilic nature of SnO_2_, arising from its abundant surface hydroxyl groups, together with the porous morphology of the catalyst layer, which promotes rapid water infiltration. To further examine whether catalyst coverage affects wettability, we measured the contact angles of R-BNP/SnO_2_ electrodes with different R-BNP loadings. Similar to the above observations, all samples exhibited instantaneous water spreading, and no discernible difference in static wettability was observed (Figure S8b). These results indicate that the coverage-dependent CO_2_RR performance is not associated with changes in macroscopic hydrophilicity or hydrophobicity.

**Figure S9**. LSV curves (a) and FEs (b) of various R-BNP/SnO_2_ catalysts with different chiral molecule loading ratios (40% stands for the ratio of R-BNP to SnO_2_ is 2:5).

The dependence of catalytic activity on BNP loading is attributed to molecular-level interfacial regulation. An intermediate R-BNP coverage (40%) provides sufficient chiral modulation to induce an effective CISS effect while maintaining adequate exposure of catalytically active Sn sites. At lower coverage (20%), the density of chiral molecules is insufficient to generate strong spin polarization, whereas excessive R-BNP loading (80%) can partially block accessible Sn active sites and introduce steric hindrance for CO_2_ adsorption and intermediate conversion, resulting in reduced catalytic performance (Figure S9). Therefore, the observed volcano-type dependence of CO_2_RR activity on R-BNP coverage arises from a balance between maximizing CISS-mediated interfacial regulation and preserving accessible catalytic sites, rather than from changes in the macroscopic wettability of electrodes.

**Figure S10.** Equivalent circuit models employed for the fitting of EIS data for SnO_2_, R-BNP/SnO_2_, S-BNP/SnO_2_, and Rac-BNP/SnO_2_.

**Figure S11.** In situ EIS measurements and corresponding phase angle vs. frequency plots for (a) SnO_2_ and (b) Rac-BNP/SnO_2_ collected over the potential range from -0.6 to -1.3 V vs. RHE.

**Figure S12.** Cyclic voltammetry (CV) curves of (a) SnO_2_, (b) R-BNP/SnO_2_, (c) S-BNP/SnO_2_, and (d) Rac-BNP/SnO_2_ recorded at scan rates ranging from 10 mV s^-1^ to 50 mV s^-1^ in 0.5 M KHCO_3_ electrolyte.

**Figure S13.** Electrochemically active surface area (ECSA) estimated from electrochemical double-layer capacitance (C_dl_) measurements for SnO_2_, R-BNP/SnO_2_, S-BNP/SnO_2_, and Rac-BNP/SnO_2_.

**Figure S14.** Pre-reaction and post-reaction high-resolution XPS spectra of P 2*p* for R-BNP/SnO_2_.

**Figure S15.** Transmitted CD spectra of R-BNP/SnO_2_, S-BNP/SnO_2_, and Rac-BNP/SnO_2_ samples after electrolysis.

**Figure S16**. ^1^H NMR spectrum of KHCO_3_ solution, R-BNP solution, electrolyte with electrode before reaction, and electrolyte with electrode after reaction.

**Figure S17**. In-situ ATR-SEIRAS spectra for (a) SnO_2_, (b) R-BNP/SnO_2_, and (c) Rac-BNP/SnO_2_ (full range).

**Figure S18**. In-situ ATR-SEIRAS spectra for (a) R-BNP/SnO_2_, and (b) Rac-BNP/SnO_2_ under positive scan (from -1.2 V to -0.6 V vs. RHE).

As shown in **Figure S18**, the characteristic intermediate bands on R-BNP/SnO_2_ gradually decrease and disappear during the reverse scan (from -1.2 V to -0.6 V vs. RHE), further confirming that these bands correspond to electrochemically generated surface intermediates rather than irreversible changes of the catalyst surface. It is worth noting that, during the reverse scan, the two closely spaced bands gradually converge into a single broadened feature. This behavior is likely associated with the potential-dependent evolution of the adsorption configuration and local environment of the surface intermediates. As the potential is swept anodically, changes in adsorbate–adsorbate interactions and vibrational frequencies lead to increased spectral overlap, making the two neighboring bands progressively unresolved. For comparison, only weak spectral evolution is observed for Rac-BNP/SnO_2_ over the same potential range, indicating substantially lower accumulation of these intermediates under identical experimental conditions. This phenomenon is consistent with our conclusion that the chiral interface modifies the stabilization and evolution of reaction intermediates, thereby influencing the reaction pathway and kinetics.

**Figure S19** Enlarged in situ ATR-SEIRAS spectra showing the characteristic vibrational regions of adsorbed *COOH and *OCHO intermediates on (a) SnO_2_, (b) R-BNP/SnO_2_, and (c) Rac-BNP/SnO_2_ electrodes.

Compared with pristine SnO_2_, the chiral R-BNP/SnO_2_ catalyst exhibits a more pronounced potential-dependent evolution of the *OCHO-related band, whereas the Rac-BNP/SnO_2_ sample shows only limited evolution of this band over the same potential range despite having an identical chemical composition. These spectral differences are consistent with the electrochemical product selectivity results and suggest that the chiral interface influences the accumulation and evolution of reaction intermediates, thereby modifying the relative energetics of competing reaction pathways.

**Figure S20.** In situ ATR-SEIRAS spectra of interfacial water on bare SnO_2_ (red: weak H-bonded water; blue: strong H-bonded water).

**Figure S21.** In situ ATR-SEIRAS spectra of interfacial water on R-BNP/SnO_2_ (red: weak H-bonded water; blue: strong H-bonded water).

**Figure S22.** In situ ATR-SEIRAS spectra of interfacial water on Rac-BNP/SnO_2_ (red: weak H-bonded water; blue: strong H-bonded water).

**Figure S23.** Ratio of peak areas for strong H-bonded water (∼3250 cm^-1^) to weak H-bonded water (∼3450 cm^-1^) over SnO_2_, R-BNP/SnO_2_, and Rac-BNP/SnO_2_.

**Table S1.** Faradaic efficiency of formate, CO, and H_2_ on SnO_2_ measured after 20 min electrolysis at various applied potentials.

| **Potential**  **(V vs. RHE)** | **FE_formate_ (%)** | **FE_CO_ (%)** | **FE_H2_ (%)** |
| --- | --- | --- | --- |
| -0.7 | 21.89 | 25.13 | 48.59 |
| -0.8 | 29.57 | 27.82 | 45.92 |
| -0.9 | 22.85 | 31.48 | 47.90 |
| -1.0 | 25.94 | 28.98 | 43.61 |
| -1.1 | 24.34 | 24.81 | 53.28 |
| -1.2 | 19.89 | 27.03 | 52.41 |

**Table S2.** Faradaic efficiency of formate, CO, and H_2_ on R-BNP/SnO_2_ measured after 20 min electrolysis at various applied potentials.

| **Potential**  **(V vs. RHE)** | **FE_formate_ (%)** | **FE_CO_ (%)** | **FE_H2_ (%)** |
| --- | --- | --- | --- |
| -0.7 | 52.44 | 21.67 | 26.48 |
| -0.8 | 45.89 | 23.17 | 25.82 |
| -0.9 | 49.45 | 19.78 | 28.65 |
| -1.0 | 48.90 | 24.78 | 29.98 |
| -1.1 | 54.75 | 19.09 | 23.78 |
| -1.2 | 57.89 | 17.62 | 22.87 |

**Table S3.** Faradaic efficiency of formate, CO, and H_2_ on S-BNP/SnO_2_ measured after 20 min electrolysis at various applied potentials.

| **Potential**  **(V vs. RHE)** | **FE_formate_ (%)** | **FE_CO_ (%)** | **FE_H2_ (%)** |
| --- | --- | --- | --- |
| -0.7 | 48.48 | 24.34 | 19.47 |
| -0.8 | 39.85 | 25.09 | 24.11 |
| -0.9 | 42.52 | 27.58 | 27.90 |
| -1.0 | 43.11 | 26.46 | 26.11 |
| -1.1 | 45.41 | 23.47 | 30.58 |
| -1.2 | 37.47 | 29.46 | 32.77 |

**Table S4.** Faradaic efficiency of formate, CO, and H_2_ on Rac-BNP/SnO_2_ measured after 20 min electrolysis at various applied potentials.

| **Potential**  **(V vs. RHE)** | **FE_formate_ (%)** | **FE_CO_ (%)** | **FE_H2_ (%)** |
| --- | --- | --- | --- |
| -0.7 | 13.32 | 29.98 | 47.98 |
| -0.8 | 15.73 | 28.98 | 45.98 |
| -0.9 | 13.56 | 29.44 | 51.53 |
| -1.0 | 16.36 | 30.15 | 46.66 |
| -1.1 | 12.49 | 22.46 | 53.74 |
| -1.2 | 14.87 | 25.37 | 56.54 |

**Table S5.** EIS fitting results for SnO_2_, R-BNP/SnO_2_, and Rac-BNP/SnO_2_ in 0.5 M KHCO_3_ electrolyte.

| **Catalysts** | **R_ct_** (kΩ) |
| --- | --- |
| SnO_2_ | 3.68 |
| R-BNP/SnO_2_ | 3.72 |
| Rac-BNP/SnO_2_ | 3.91 |

**References**

62. Henderson, J. D.; Pearson, L.; Nie, H. Y.; Biesinger, M. C. “X-Ray photoelectron spectroscopy analysis of indium and indium-containing compounds,” *Surf. Interface Anal.* 57 (2024): 81-97.
